# Episcleral eye plaque dosimetry comparison for the Eye Physics EP917 using Plaque Simulator and Monte Carlo simulation

**DOI:** 10.1120/jacmp.v16i6.5659

**Published:** 2015-11-08

**Authors:** Leonard W. Zimmermann, Ahmad Amoush, Douglas A. Wilkinson

**Affiliations:** ^1^ Department of Physics Cleveland State University Cleveland OH; ^2^ Department of Radiation Oncolog Georgia Regents University Augusta GA; ^3^ Department of Radiation Oncology Cleveland Clinic Cleveland OH USA

**Keywords:** eye plaque brachytherapy, dosimetry, Monte Carlo, ${\;}^{125}I$

## Abstract

This work is a comparative study of the dosimetry calculated by Plaque Simulator, a treatment planning system for eye plaque brachytherapy, to the dosimetry calculated using Monte Carlo simulation for an Eye Physics model EP917 eye plaque. Monte Carlo (MC) simulation using MCNPX 2.7 was used to calculate the central axis dose in water for an EP917 eye plaque fully loaded with 17 IsoAid Advantage ${\;}^{125}I$ seeds. In addition, the dosimetry parameters Λ, gL(r), and F(r,θ) were calculated for the IsoAid Advantage model IAI‐125 ${\;}^{125}I$ seed and benchmarked against published data. Bebig Plaque Simulator (PS) v5.74 was used to calculate the central axis dose based on the AAPM Updated Task Group 43 (TG‐43U1) dose formalism. The calculated central axis dose from MC and PS was then compared. When the MC dosimetry parameters for the IsoAid Advantage ${\;}^{125}I$ seed were compared with the consensus values, Λ agreed with the consensus value to within 2.3%. However, much larger differences were found between MC calculated gL(r) and F(r,θ) and the consensus values. The differences between MC‐calculated dosimetry parameters are much smaller when compared with recently published data. The differences between the calculated central axis absolute dose from MC and PS ranged from 5% to 10% for distances between 1 and 12 mm from the outer scleral surface. When the dosimetry parameters for the ${\;}^{125}I$ seed from this study were used in PS, the calculated absolute central axis dose differences were reduced by 2.3% from depths of 4 to 12 mm from the outer scleral surface. We conclude that PS adequately models the central dose profile of this plaque using its defaults for the IsoAid model IAI‐125 at distances of 1 to 7 mm from the outer scleral surface. However, improved dose accuracy can be obtained by using updated dosimetry parameters for the IsoAid model IAI‐125 ${\;}^{125}I$ seed.

PACS number: 87.55.K‐

## I. INTRODUCTION

Choroidal melanoma is the most common primary intraocular tumor among adults, and eye plaque brachytherapy is a technique used to treat ocular melanoma as an alternative to total removal of the eye.^(1)^ The Collaborative Ocular Melanoma Study (COMS) was created as a multi‐institutional cooperative clinical trial sponsored by the National Eye Institute of the National Institutes of Health (Bethesda, MD).^(2)^ The COMS group standardized methods of plaque brachytherapy for choroidal melanomas and found through randomized clinical trials that brachytherapy with ${\;}^{125}I$ eye plaques was as effective as enucleation for medium‐sized choroidal melanomas, with the added advantages of eye and vision preservation.^(3)^

The use of ${\;}^{125}I$ radioactive source eye plaques is the most common treatment modality based on practical considerations and scientific reasons as recommended by (COMS).^4^, ^5^, ^6^ Recent updates of the brachytherapy dosimetry guidelines, such as the Task Group 43 update (TG43‐U1) and supplement (TG‐43U1S1), published in 2004 and 2007 respectively, have addressed concerns regarding dose calculations at distances below 1.0 mm, from low‐energy brachytherapy sources.^7^, ^8^ However, the AAPM recommendations in the TG‐43U1 and TG‐43U1S1 reports are limited to infinite homogeneous water medium and do not account for the effects of material heterogeneities, such as the eye plaque and bony orbit, which are of higher effective atomic number $Z_{\text{eff}}$ than water. AAPM Task Group 129^(9)^ reviewed the dosimetry of eye plaque brachytherapy and evaluated the impact of heterogeneity effects for ${\;}^{125}I$ and ${\;}^{103}\text{Pd}$ COMS plaques only; they then made recommendations for treatment planning and quality assurance for eye‐plaque brachytherapy. Since the scope of AAPM TG 129 was limited to COMS plaques, there is a need for further research into the heterogeneity effects of other plaques.

Plaque Simulator (PS) (Eye Physics, LLC, Los Alamitos, CA) is a dedicated treatment planning system for eye plaque brachytherapy. Eye plaque therapy involves treating the choroidal melanomas with small radioactive sources embedded in a plaque which is placed adjacent to the tumor on the outer scleral surface. PS is based on superposition of dose contributions from individual seeds following the TG‐43 formalism.^(9)^ The software incorporates additional correction factors, such as plaque shell collimation, slot collimation, and carrier attenuation, that allow corrections for source attenuation and scatter.^(10)^ An alternative and more accurate way to check the dosimetry of the eye plaque is Monte Carlo (MC) simulation. Monte Carlo N‐Particle eXtended (MCNPX 2.70) MC code,^(11)^ developed by Los Alamos National Laboratory, was used in this study to calculate the energy deposited in water as recommended by TG‐43U1.

The Eye Physics Model EP917 eye plaque, with 17 seeds in 0.8 mm deep slots, has no published dosimetric data using the IsoAid Advantage ${\;}^{125}I$ seed. The only published data for the EP917 were obtained with the Amersham‐Health model 6711 ${\;}^{125}I$ seed using diodes and has never been verified with MC simulation.^(12)^ A method has been described for verification that the activity or air kerma strength of preassembled eye plaques agrees with the activity or air kerma strength called for in the treatment plan; however, this method is not a dosimetric comparison.^(13)^

The primary goal of this study was to calculate the central axis dose (CAX) for a fully loaded EP917, using both PS and MC, and to compare the results. For this study, the plaque seed placement from PS was recreated in the MC model to ensure that both were identical. The actual geometry of the model EP917 plaque was not verified in this study, but work presented by Aryal et al.^(14)^ indicates that differences have been observed between the EP917 plaque geometry and its PS model. Since this study is a comparison between MC and PS calculated CAX dose, the same plaque geometry is used in both, and the accuracy of the physical plaque geometry is not pertinent to this study.

A secondary goal of this study was to develop the MCNP model for the IsoAid Advantage ${\;}^{125}I$ seed and verify its dosimetric parameters against its consensus values. The default dosimetric parameters for the IsoAid Advantage IAI‐125 seed were then replaced in PS with the values calculated in our study and the CAX dose was recalculated.

## II. MATERIALS AND METHODS

A multiple step process was employed to accurately compare the CAX from MC simulation to the CAX calculated by PS. First, the IsoAid Advantage 125I seed (model IAI‐125) (IsoAid LLC, Port Richey, FL) was modeled in MCNPX and its dosimetric characteristics were benchmarked. Next, the EP917 plaque, including the seed slots, was modeled and the seeds were placed in their proper locations within the plaque. The positions and dimensions of the seed slots and the seeds within the plaque were derived from PS, as described later in the Eye Physics EP917 Characteristics section (Material & Methods B below). Seventeen MCNPX input files were then created for the EP917 with a single seed in each of the 17 possible locations. Next, the Monte Carlo simulation was run using $4\times 10^{8}$ particle histories with each of the seventeen input files. The results of each particle history, a track length estimate of photon energy deposition, were accumulated or tallied. This method was chosen in order to facilitate debugging of the input files. Finally, the MC CAX results were calculated and combined for comparison with PS. In parallel, dose table reports containing CAX information were created in PS for the same 17 single‐seed placements used in MC. The CAX data from the dose table reports were then combined and compared with the Monte Carlo results.

### A. IsoAid Advantage ${\;}^{125}I$ (model IAI‐125) source characteristics

The IsoAid Advantage ${\;}^{125}I$ (model IAI‐125) brachytherapy seed is a 0.8 mm diameter, 4.5 mm long titanium capsule containing a 3 mm long ${\;}^{125}I$ source. The ${\;}^{125}I$ source is a 0.5 mm diameter silver rod, 3 mm long with a 1 μm thick coating of silver iodide (AgI) adsorbed onto its surface.^(15)^

**Figure 1:**
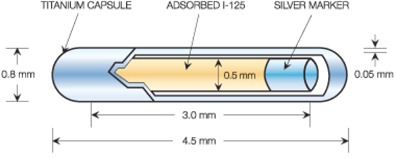
Diagram showing the dimensions of the IsoAid Advantage ${\;}^{125}I$ seed used in this study.^(15)^

The seed capsule was modeled in MCNPx by a 0.8 mm outside diameter titanium cylinder with a 0.05 mm thick wall. The seed ends were formed by 0.8 mm outside diameter hemispheres placed at either end of the cylinder. To create the end welds with a maximum thickness of 0.1 mm, 0.7 mm diameter hemispheres were placed inside the larger hemispheres at the ends of the cylinder. These hemispheres were offset towards the center of the seed by 0.05 mm to give the end welds a maximum thickness of 0.1 mm. The volume between the seed and the outer capsule was filled with NIST dry air. The AgI source coating was created in the MCNPX simulation by surrounding the silver cylinder with an AgI cylinder that is 1 μm larger in radius and 2 μm longer (1 μm longer at each end). The AgI coating is defined by the volume between the two cylinders. The dimensions used in this model are the same as those used in Meigooni et al.^(16)^ and Taylor and Rogers^(17)^ (Fig. 1).

#### A.1 Air‐kerma strength (SK)

The air‐kerma strength (SK), in units of $U(U={\text{cGycm}}^{2}h^{-1})$, of the IsoAid Advantage ${\;}^{125}I$ seed was calculated in a 0.02 cm×0.02 cm×5 cm radius scoring ring centered on the transvers axis of the seed in a vacuum, as shown in Eq. (1). The photon spectrum from the source is produced isotopically and uniformly so the scoring cell in this study can be approximated as a point scoring voxel.

(1) $$S_{K}(cGycm^{2}h^{-1})=F6:P\left(\frac{cGy}{photon}\right)*1.5767\left(\frac{photons}{decay}\right)*3600\left(\frac{\text{sec}}{h}\right)*d^{2}(cm)$$

where $F6:P\left(\frac{cGy}{photon}\right)$ is the MCNP tally result in $\text{cGy}/\text{photon},1.5767\frac{photons}{decay}$ is the photon yield per decay for ${\;}^{125}I$ from the NNDC decay spectrum,^(18)^ and *d* is the radial distance from the center of the source.

#### A.2 Dose rate constant (Λ)

The dose rate constant (Λ) was calculated, as shown in Eq. (2), by dividing the dose to water in a 0.02 cm×0.02 cm×1 cm radius scoring ring centered on the transvers axis of the seed at the reference position, $D(1\;\text{cm},\;90^{\circ })$, within a 30 cm radius water filled sphere, by the air‐kerma strength (SK).

(2) $$\Lambda (cGyh^{-1}U^{-1})=\frac{1}{S_{k}}(U^{-1})*F6:P\left(\frac{cGy}{photon}\right)*1.5767\left(\frac{photons}{decay}\right)*3600\left(\frac{\text{sec}}{h}\right)$$

where $F6:P\left(\frac{cGy}{photon}\right)$ is the MCNP tally result in $\text{cGy}/\text{photon},1.5767\frac{photons}{decay}$ is the photon yield per decay for ${\;}^{125}I$ from the NNDC decay spectrum.^(18)^

#### A.3 Radial dose function gL(r)

To calculate the radial dose function, the source was positioned at the center of a 30 cm radius water filled sphere and an array of scoring rings was defined within this sphere. The rings were defined by concentric pairs of cylinders aligned with the seed's long axis. The 0.2 mm height of rings was defined between a pair of planes parallel to the seed's transvers axis, 0.1 mm above and below the seed center. Radial dose function was determined at distances ranging from 0.1 to 10 cm from the center of the source at the distances shown in Table 1.

**Table 1:** gL(r), for the IsoAid model IAI‐125 ${\;}^{125}I$ seed from this study, consensus values from TG‐43U1S1, and recently published values from Taylor and Rogers^(17)^ and Aryal et *al*.^(26)^

*R (cm)*	*0.1*	*0.2*	*0.3*	*0.5*	*0.6*	*0.7*	*1*	*2*	*3*	*4*	*5*	*7*	*9*	*10*
This study	1.072	1.095	1.092	1.074	1.061	1.046	1.000	0.817	0.637	0.482	0.362	0.196	0.106	0.078
TG‐43U1S1	1.040	‐	‐	1.080	‐	‐	1.000	0.800	0.611	0.468	0.368	0.227	0.141	0.090
Taylor and Rogers^(17)^	1.080	1.099	1.096	1.076	1.064	1.052	1.003	0.819	0.636	0.484	0.367	0.200	0.107	0.080
Aryal et al.^(26)^	‐	‐	‐	1.073	‐	‐	0.999	0.814	0.635	0.484	0.364	0.200	0.109	0.080

#### A.4 2D anisotropy function F(r,θ)

The 2D anisotropy function was calculated with the source positioned at the center of a 30 cm radius water‐filled sphere. An array of scoring cells was formed using spheres and ring toruses. The axis of revolution of each torus was perpendicular to the source's long axis. The centers of the spheres and torus major axis radii were placed at radial distances ranging from 0.5 to 7 cm from the center of the source at the distances shown in Table 2. The torus major axis radii were placed at radial angles of 5° and from 10° to 90° from the long axis of the source in 10° increments. Spherical scoring cells were placed along the axis of the source (0°). The volume of the scoring cells was increased with increasing distance from the center of the source by setting the radii of the spheres and torus minor axes to 2% of the radial distance to the source.

**Table 2:** F(r,θ) for the IsoAid model IAI‐125 ${\;}^{125}I$ seed from this study

	*r (cm)*					
*Theta °*	*0.5*	*1*	*2*	*3*	*5*	*7*
0	0.311	0.374	0.481	0.562	0.655	0.693
5	0.547	0.597	0.643	0.684	0.724	0.750
10	0.561	0.612	0.673	0.712	0.746	0.771
20	0.754	0.775	0.806	0.823	0.843	0.852
30	0.884	0.884	0.895	0.902	0.909	0.910
40	0.963	0.954	0.952	0.953	0.952	0.952
50	1.009	0.999	0.991	0.988	0.982	0.981
60	1.031	1.026	1.015	1.011	1.003	0.996
70	1.032	1.039	1.029	1.024	1.014	1.010
80	0.995	1.029	1.032	1.028	1.018	1.014
90	1	1	1	1	1	1

### B. Eye Physics EP917 characteristics

The Eye Physics EP917 is a 16×14 mm semi‐elliptical plaque with a broad notch in its posterior edge and two suture eyelets at its anterior edge.^(19)^ It is made of 18 karat gold alloy and is nominally 1.5 mm thick.^(19)^ There are 17 seed positions in 0.8 mm deep slots in a semi‐elliptical seed pattern; the seeds are glued into slots using cyanoacrylate or dental acrylic. The slots in the plaque collimate each radiation source to remove laterally directed primary radiation that does not contribute to the tumor dose.^(19)^

**Figure 2:**
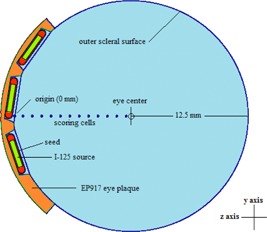
Diagram showing a side view of the MCNPX model of the EP917 plaque, IsoAid Advantage ${\;}^{125}I$ seeds, and the dose scoring cells. This view is along horizontal x‐axis, looking at a plane on the y‐ and z‐axes, through the origin of the plaque.

The EP917 plaque shell is made of 18K Yellow Standard gold alloy consisting of 75% gold and 25% silver and copper.^(20)^ 18 Karat Yellow Gold consists of 75% gold, 15% copper, and 10% silver.^(21)^ These are the proportions by mass of gold, silver, and copper used in the MCNPx plaque model. In MCNPx, the eye is modeled as a water‐filled, 12.5 mm radius sphere and the EP917 lies on the surface of this sphere (see Fig. 2).

**Figure 3:**
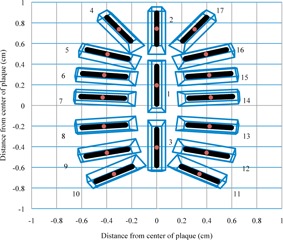
Excel chart showing the X and Y coordinates of the seed ends, plaque slots, and seed centers.

PS uses a spherical coordinate system to define the location of each seed within the plaque.^(22)^ Four parameters, Alpha, Beta, Tilt, Offset, and radius from the origin locate the center of each seed within the plaque, its rotation about its center, and its distance from the origin. The origin in this coordinate system is the center of a sphere defined by the radius of curvature of the inner surface of the plaque. In order to be modeled in MCNPX 2.7, the seed positions were converted from the PS coordinates to Cartesian coordinates. The seed slots in the plaque were modeled using arbitrary polyhedrons. The coordinates of the corners of the polyhedrons were calculated from the alpha and beta angles and the slot face and back dimensions from the PS slot editor (Fig. 3).

The model of the loaded EP917 was verified prior to MC simulation using several methods. The seed centers (Table 3), and the source and seed end positions (Table 4) were verified by comparison to the Cartesian coordinates listed in the Slot Editor in PS for each seed. The seed slot face and back corners were checked for coplanarity to ensure that they lie in the same plane and are at the same radial distance from the origin (Table 3).

It has recently been reported that the slot dimensions in PS do not accurately reflect the physical geometry of the model EP917 plaque.^(14)^ The accuracy of the physical EP917 slot dimensions is not relevant to this study, as it is a comparison between MC and PS utilizing the same plaque and slot geometry.

Finally, 13 dose scoring cells were created using 0.1 mm radius water‐filled spheres centered on the z‐axis at 1 mm spacing, beginning at the external scleral surface and extending through the origin. The first cell, at 0 mm, was excluded from this study since it is centered on the outer scleral surface, half of its volume is within the gold alloy plaque and, as a result, it is partially shielded from neighboring sources (Fig. 2).

**Table 3:** Cartesian coordinates of the seed centers calculated from the PS coordinates (Alpha, Beta, Tilt, and Offset) and the radius from the plaque's center of curvature, 1.25 cm

	*PS Coordinates*				*Cartesian Coordinates (cm)*		
				*Offset*			
*Seed No.*	$Alpha^{\circ }$	$Beta^{\circ }$	$Tilt^{\circ }$	*(cm)*	*X*	*Y*	*Z*
1	8.7	0	0	0.04	0.000	0.195	1.275
2	35.2	0	0	0.04	0.000	0.744	1.054
3	18.4	180	0	0.04	0.000	−0.407	1.224
4	38.2	22.3	14	0.04	0.303	0.738	1.014
5	29.4	38.4	33	0.04	0.393	0.496	1.124
6	23.4	55	28	0.04	0.420	0.294	1.184
7	19.9	79.8	7	0.04	0.432	0.078	1.213
8	21.3	115.6	160	0.04	0.423	−0.202	1.202
9	28.1	139	146	0.04	0.399	−0.459	1.138
10	35.4	152.8	149	0.04	0.342	−0.665	1.052
11	35.4	207.2	31	0.04	−0.342	−0.665	1.052
12	28.1	221	34	0.04	−0.399	−0.459	1.138
13	21.3	244.4	20	0.04	−0.423	−0.202	1.202
14	19.9	280.2	173	0.04	−0.432	0.078	1.213
15	23.4	305	152	0.04	−0.420	0.294	1.184
16	29.4	321.6	147	0.04	−0.393	0.496	1.124
17	38.2	337.7	166	0.04	−0.303	0.738	1.014

**Table 4:** Cartesian coordinates of the inner and outer seed ends relative to the center of the radius of curvature of the plaque, 1.25 cm

	*Seed Inner End Coordinates (cm)*			*Seed Outer End Coordinates (cm)*		
*Seed #*	*x1*	*y1*	*z1*	*x2*	*y2*	*z2*
1	0.000	0.418	−1.241	0.000	−0.027	−1.309
2	0.000	0.927	−0.924	0.000	0.560	−1.184
3	0.000	−0.621	−1.153	0.000	−0.194	−1.295
4	0.418	0.876	−0.879	0.187	0.600	−1.149
5	0.592	0.549	−1.031	0.195	0.444	−1.216
6	0.630	0.312	−1.105	0.210	0.276	−1.263
7	0.644	0.088	−1.137	0.221	0.068	−1.289
8	0.212	−0.187	−1.279	0.633	−0.218	−1.125
9	0.196	−0.417	−1.226	0.602	−0.500	−1.050
10	0.167	−0.578	−1.163	0.517	−0.751	−0.940
11	−0.517	−0.751	−0.940	−0.167	−0.578	−1.163
12	−0.602	−0.500	−1.050	−0.196	−0.417	−1.226
13	−0.633	−0.218	−1.125	−0.212	−0.187	−1.279
14	−0.221	0.068	−1.289	−0.644	0.088	−1.137
15	−0.210	0.276	−1.263	−0.630	0.312	−1.105
16	−0.195	0.444	−1.216	−0.592	0.549	−1.031
17	−0.187	0.600	−1.149	−0.418	0.876	−0.879

### C. Monte Carlo simulation

Monte Carlo simulation using MCNPX 2.7 was used for this study. In all cases, the NNDC decay spectrum was used.^(18)^ The MCNP F6:P tally, which is a running total of the photon energy deposited within a scoring cell volume, was used to accumulate histories as electron equilibrium was assumed.^(23)^ The F6:P tally calculates the average absorbed energy, assuming all the secondary particles are absorbed locally.^(11)^

For this study, a single seed with air‐kerma strength 4.247 U was placed in one of the 17 seed locations in the EP917 and the CAX dose was calculated using MC simulation. This was repeated 17 times, once for each seed location in the EP917. The CAXs from all 17 MC simulations were then summed to arrive at the CAX dose for an EP917 containing all 17 seeds. This yields a dose of approximately 85 Gy at the prescription point, 5 mm from the inner scleral surface (6 mm from the outer surface).

An energy deposition tally in units of MeV/g (F6:P tally) with a tally multiplier of $1.602E^{10}$ was used to calculate the dose in Gy averaged over a cell ($1\;\text{MeV}/g=1.602\times 10^{10}\;\text{Gy}$) using Eq. (3).^(24)^

(3) $$Dose(Gy)=F6:P\left(\frac{cGy}{photon}\right)*1.5767\left(\frac{photon}{decay}\right)*\frac{4.247U}{S_{Kmc}}\left(\frac{decay}{\text{sec}}\right)*3600\left(\frac{\text{sec}}{h}\right)*72(h)*(1/100)\left(\frac{Gy}{cGy}\right)$$

where $F6:P\left(\frac{cGy}{photon}\right)$ is the MCNP tally result in $\text{cGy}/\text{photon},1.5767\frac{photons}{decay}$ is the photon yield per decay for ${\;}^{125}I$ from the NNDC decay spectrum.^(18)^

### D. Plaque Simulator (PS) treatment planning

Bebig Plaque Simulator (Eye Physics, LLC) is a 3D treatment simulation and modeling package for plaque therapy of ocular tumors and macular degeneration. PS predicts the dose based on AAPM TG‐43U1 dose formalism; single factors are used to account for source collimation and backscatter from the gold alloy plaque.

For dose calculations, PS uses extended gL(r) and F(r,θ) lookup tables based upon the values published in AAPM TG‐43U1S1.^(8)^ The dose rate constant ($\text{cGy}\;h^{-1}U^{-1}$) default values are taken from the Table 1 of TG‐43U1S1. A scatter modifier function B(r) is used to compensate for deviation from homogeneous full scatter geometry.^(10)^ The dose calculation options used in this study were standard calculation, anisotropy, linear sources, scatter modifier B(r), partial exposure, and slot collimation. These are shown in the PS reports as "Dose calc. mode: Standard, Line, F[ϕ],B[r], Pexp, Slot".

As with the MC simulation, a single seed with air‐kerma strength 4.247 U was placed in one of the 17 seed locations in the EP917 and the dose distribution was calculated. This was repeated 17 times, once for each seed location in the EP917. The duration of dose integration was 72 hrs with a prescription dose of 85 Gy at 6 mm with a single seed at seed position 1.

## III. RESULTS

### A. IsoAid Advantage ${\;}^{125}I$ (model IAI‐125)

#### A.1 Dose rate constant Λ

For the IsoAid Advantage ${\;}^{125}I$ seed, the calculated dose rate constant of $0.958\pm 0.003\;\text{cGy}\;h^{-1}U^{-1}$ agrees with the consensus value from $\text{TG}-43U1S1,0.981\pm 0.003\;\text{cGy}\;h^{-1}U^{-1}$, to within 2.3%.^(8)^ However, Λ from this study agrees with MC and TLD results reported by Solberg et al.^(25)^ within 0.4 and 0.2% respectively and to within 0.1% of recent work by Taylor and Rogers,^(17)^ using point voxel scoring cells. When compared with Λ, calculated using WAFAC voxel scoring cells, Λ from this study is 3.6% higher.^(17)^ When compared with Λ from recently published work by Aryal et al.^(26)^ using WAFAC voxel scoring cells and slightly different seed design (simulation condition 11), Λ from this study is 3.9% higher (Table 5).

**Table 5:** Dose rate constant comparison between results from this study and recently published literature. Aryal et al.^(26)^ present results using 12 different simulation conditions; simulation condition 11 is assumed to best represent the IsoAid model IAI‐125 ${\;}^{125}I$ seed

*Authors*	*Dose Rate Constant (Λ)*	*Percent Difference From Published Literature*
This work	0.958±0.003	‐
Consensus value^(8)^	0.981	−2.3%
Solberg et al.^(25)^		
MCNP, version 4C	0.962±0.005	−0.4%
TLD in Plastic Water	0.96±0.05	−0.2%
Taylor and Rogers^(17)^		
EGSnrc user‐code BrachyDose – WAFAC	0.925±0.002	3.6%
EGSnrc user‐code BrachyDose – Point	0.959±0.002	−0.1%
Aryal et al.,^(26)^ MCNPX, (simulation condition 11)	0.922	3.9%

#### A.2 Radial dose function gL(r)

The consensus values from the AAPM/IROC‐Houston Seed Registry for the radial dose function agreed with the calculated values in this work to within less than ±0.4% from 0.5 to 5 cm, but above 5 cm the percent error increased, reaching a maximum of 29% at 9 cm (Table 1).

**Figure 4:**
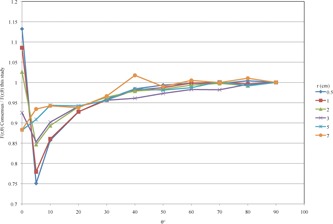
Ratio of gL(r) from published literature to gL(r) from this study.

When compared with more recent published data from Taylor and Rogers^(17)^ and Aryal et al.,^(26)^ the radial dose function values in this work agree to within ±2% over the entire range from 0.5 to 10 cm (Fig. 4).

#### A.3 2D anisotropy function F(r,θ)

The anisotropy function results from this study, calculated using arrays of scoring cells formed from spheres along the seed axis $\left(\theta =0^{\circ }\right)$ and ring toruses for all $\theta >0^{\circ }$, are shown in Table 2.

**Figure 5:**
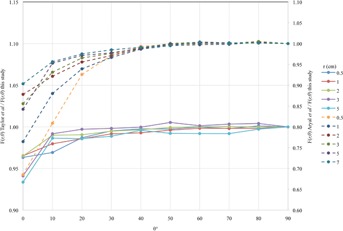
The ratio F(r,θ) consensus / F(r,θ) this study.

The consensus values from the AAPM/IROC‐ Houston Seed Registry for the 2D anisotropy function agree with this work to within ±5% for angles greater than 30°; however, at angles less than 30°, the percent error increases; at 0.5 cm at 0°, the error is 13.2% and at 0.5 cm at 5°, the error is −24.9% (Fig. 5).

**Figure 6:**
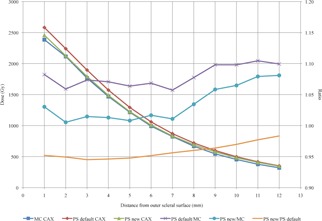
The ratio F(r,θ) Taylor and Rogers^(17)^ (solid line) and Aryal et al.^(26)^ (dashed line) / F(r,θ) this study.

When compared with recently published data from Taylor and Rogers,^(17)^ agreement is between −3.7% and 0.5% for all points, except at 3 and 5 cm at 0°, at which these points disagree by −5.9% and ‐6.6%, respectively. When compared with recently published data from Aryal et al.,^(26)^ agreement is between −3.3% and 0.5% for all points at angles $\geq 30^{\circ }$. The differences increase for angles $<30^{\circ }$, reaching maximums at 0° (Fig. 6).

### B. Plaque dosimetry

The difference between MC and PS calculated CAX ranged from a minimum of 5.7% to a maximum of 10.4% at distances from 1 mm to 12 mm from the outer scleral surface. Between 1 and 7 mm from the outer scleral surface the average difference is 6.8%, but increases to an average of 10% between 9 and 12 mm from the outer scleral surface.

**Figure 7:**
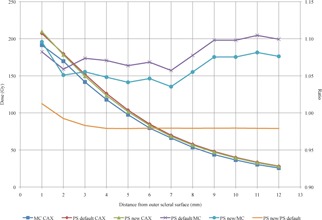
The CAX dose calculated by MC and PS with default and new dosimetric parameters from this study for the IsoAid Advantage IAI‐125 ${\;}^{125}I$ seed. The ratios of PS to MC CAX dose for PS with both default and new dosimetric parameters, and the ratio of PS CAX with new/default dosimetric parameters showing the reduction in CAX dose.

When Λ, gL(r), and F(r,θ) from this study are substituted for the default consensus values for the IsoAid model IAI‐125 in PS, the difference between the MC and PS CAX dose to is reduced by 2.2% from 4 to 12 mm from the outer scleral surface. This is likely due to the 2.3% lower Λ from this study, as it shows a linear dose decrease. However, at distances between 1 and 4 mm from the outer scleral surface, the difference changes nonlinearly. At 1 mm, the deviation between the PS and MC CAX dose increases to 9.6% but decreases 4.1% at 4 mm. This nonlinear change between 1 and 4 mm is likely due to the differences between gL(r) and F(r,θ) from this study and the PS default consensus values (Fig. 7, Table 6).

**Table 6:** MC and PS calculated CAX depth doses at distances from the outer scleral surface. For this study, EP917 was fully loaded with 17 IsoAid Advantage ${\;}^{125}I$ seeds with an AKS of 4.247 U. The irradiation time was 72 hrs, resulting in a dose of 85 Gy to the prescription point located along the CAX, 6 mm from the outer scleral surface

*Distance (mm)*	*MC CAX (Gy)*	*PS Default CAX (Gy)*	*PS New CAX (Gy)*
1	191.4	207.1	209.7
2	169.7	179.7	178.4
3	141.7	152.1	149.5
4	117.9	126.2	123.5
5	97.5	103.8	101.6
6	79.6	85.0	83.2
7	66.1	69.8	68.4
8	53.6	57.7	56.5
9	43.7	48.0	47.0
10	36.4	40.0	39.1
11	30.3	33.4	32.7
12	25.6	28.2	27.6

### C. Uncertainty analysis

AAPM TG‐43U1 recommends inclusion of uncertainty analyses, specific to the methodology employed in the work, in published articles.^(27)^ Uncertainties shown in this study are statistical uncertainties only. A more detailed MC uncertainty analyses for the IsoAid model IAI‐125 may be found in the work by Aryal et al.^(26)^

The MC CAX dose uncertainties at distances of 1, 5, and 10 mm are shown in Table 6. Volume averaging was calculated by recalculating the dose using a scoring cell of 0.01 mm radius and comparing it to the dose calculated using a 0.1 mm radius scoring cell (Table 7).

**Table 7:** Generic uncertainty analysis of MCNPX simulation for 1σ

*Component of Uncertainty*	*r = 1 mm*	*r = 5 mm*	*r = 10 mm*
MCNPX Statistics (Type A)	1.1%	1.1%	2.2%
Cross Section (Type B)^(7)^	2.3%	2.3%	2.3%
Volume Averaging (Type B)	0.69%	0.50%	1.5%
Total Uncertainty	3.27%	3.23%	4.01%

## IV. DISCUSSION

### A. IsoAid Advantage ${\;}^{125}I$ (model IAI‐125)

The dose rate constant from this study is 2.3% lower than the consensus value from TG‐43U1S1, but agrees well with recently published results by Taylor and Rogers et al.^(17)^ to within 0.1% (Table 3). The scoring cells used in this study for dose to water at 1 cm and air kerma strength in vacuo at 5 cm were 0.02 cm high, 0.02 cm deep rings, this is smaller than the point voxel scoring cells used by Taylor and Rogers (0.1×0.1×0.05 cm for air‐kerma strength in vacuo and 0.01×0.01×0.01 cm for dose to water). Solberg et al.^(25)^ calculated Λ based upon air‐kerma strength calculated in a small voxel 50 cm from the source 25. As expected, Λ from this study is comparable to the point voxel‐based Λ. When compared with the Λ from Aryal et al.^(26)^ using simulation condition 11 (0.5 μm AgI coating thickness, 0.35 mm end weld thickness) and a WAFAC voxel scoring cell, Λ from this study is 3.9% higher. This is consistent with the 3.6% difference observed by Taylor and Rogers between Λ calculated using point and WAFAC scoring voxels. Also, Lymperopoulou et al.^(28)^ and Taylor et al.^(29)^ observed 3.6% and 3.3% differences between Λ calculated using point and WAFAC scoring voxels for Amersham Health Model 6711 and Tech Medical Model STM1251, respectively.

The consensus gL(r) values differed considerably from those in this study, the difference ranged from ‐4.1% at 3 cm to 33.2% at 9 cm. When compared with more recent work by Taylor and Rogers^(17)^ and Aryal et al.,^(26)^ the agreement is much better ranging from −0.1% to 1.9% and −0.2% to 4.8%, respectively. The difference between the consensus gL(r) values and recent work, including this study, is primarily due to the different cross‐section libraries and photon energy spectra used, as described by Aryal et al.^(26)^ The outdated photoelectric cross sections used by Solberg et al.^(25)^ were the XCOM tabulation of Berger and Hubbell^(30)^ and the ${\;}^{125}I$ decay spectrum was taken from that of Attix.^(31)^ This study used the MCNP default 04p cross‐section library, based upon ENDF/B‐VI Release 8 Photoatomic Data,^(32)^ and the NNDC decay spectrum.^(18)^

The anisotropy function did not agree well with consensus values, but had much better agreement with the more recent published data by Taylor and Rogers.^(17)^ The differences between our data and the consensus published data are due to different seed design parameters used in MC, specifically differences in the end weld thickness. As with the radial dose function, the energy spectrum and photon cross‐section libraries influence the results as well.^(26)^ The model IAI‐125 seed design parameters used in this study are the same as those used by Taylor and Rogers^(17)^ and Meigooni et al.^(16)^ with a maximum capsule end weld thickness of 0.1 mm and a 1 μm thick AgI source coating. The end weld thickness and source AgI coating thickness used by Solberg et al.^(25)^ were 0.24 mm and 0.5 μm, respectively. When compared to recent work by Aryal et al.,^(26)^ there is good agreement at all radial distances for $\theta \geq 30^{\circ }$, but for $\theta <30^{\circ }$, the difference increases reaching a maximum of −31.5% at a radial distance of$0.5\;\text{cm at}\;\theta =0^{\circ }$. The F(r,θ) differences between this study and that of Aryal and colleagues are due to the difference in seed capsule end weld thickness, 0.35 mm and 0.1 mm, respectively, and AgI source coating, 0.5 μm and 1 μm thick, respectively. Both studies utilized the same MCNP 04p cross‐section library^(32)^ and NNDC decay spectrum.^(18)^

### B. Plaque dosimetry

Dosimetric verification of PS with the University of Southern California (USC) #9 eye plaque (later renamed EP917) was performed by Knutsen et al.^(12)^ in 2001. For their study, the USC #9 was loaded in a nine seed configuration, using the Amersham‐Health model 6711 ${\;}^{125}I$ source. CAX measurements were made using a p‐n junction diode in a water phantom and compared to the CAX dose calculated by PS. The measured and calculated CAX depth doses were compared, and deviations up to 4% (relative to the peak dose at 2 mm) were found. When the CAX depth doses from this study were also normalized to the dose at 2 mm, the maximum difference between MC and PS CAX dose was 4.3%. However, the data are inconclusive as the measured CAX depth dose from the Knutsen study was slightly higher that the CAX depth dose from PS, and the MC CAX depth dose is lower than the PS CAX depth dose.

The difference between the PS‐calculated central axis absolute dose and that from this MC simulation ranged from 5.7% to 10.5% at distances from 1 mm to 12 mm from the outer scleral surface when using default consensus dosimetric parameters for the IsoAid model IAI‐125 ${\;}^{125}I$ seed. When the calculated dosimetric parameters from this study were substituted for the default consensus values in PS, the deviation between the PS and MC CAX depth dose decreased linearly by 2.2%, from 4 to 12 mm, with a range from to 3.5% to 8.1%. This linear decrease in CAX dose agrees well with the use of the 2.3% lower Λ from this study. However, at 1 mm from the outer scleral surface, the deviation between the PS and MC CAX dose increased by 1.4% to reach 9.6%. The difference in the PS CAX depth dose is seen in Fig. 7 as the ratio between PS new and PS default, where PS new is the CAX dose calculated with dosimetric parameters for the IAI‐125 from this study, and PS default is the CAX dose calculated with PS default parameters for the IAI‐125. The nonlinear change in the difference between PS and MC CAX dose between 1 and 4 mm may due to the differences between gL(r) and F(r,θ) from this study and the PS default consensus values. Additionally, this may also be due to the interpolation used to create the extended gL(r) and F(r,θ) lookup tables.

## V. CONCLUSIONS

The dosimetric parameters of the IsoAid model IAI‐125 obtained in this study using current cross‐section libraries and source spectrum agree well with recently published data. Some of the older consensus data should be reviewed and where necessary updated. The differences are likely due to the source design used in the previous studies, outdated cross‐section libraries and source spectrum.

Comparing the CAX depth dose from PS with the calculated CAX dose from MC showed that PS overestimates the CAX depth dose from approximately 4% to 10% for distances between 1 and 12 mm from the outer scleral surface. When the dosimetric parameters from this study Λ, gL(r), and F(r,θ)) are used in PS in place of the consensus parameters, agreement for the CAX depth dose of the EP917 plaque between PS and our MC study is improved by 2.3% from depths of 4 to 12 mm from the outer scleral surface. We conclude that PS adequately models the central dose profile of this plaque using its defaults for the IsoAid model IAI‐125, but improved dose accuracy can be obtained using updated dosimetry parameters for the IsoAid IAI‐125 ${\;}^{125}I$ seed.

## Supporting information

Supplementary Material

Supplementary Material

Supplementary Material

Supplementary Material
