# Supplementary figures and images for: Episcleral eye plaque dosimetry comparison for the Eye Physics EP917 using Plaque Simulator and Monte Carlo simulation
Source: J Appl Clin Med Phys. 2015 Nov 8;16(6):226–39. doi: 10.1120/jacmp.v16i6.5659 (PMC5691011; doi:10.1120/jacmp.v16i6.5659)

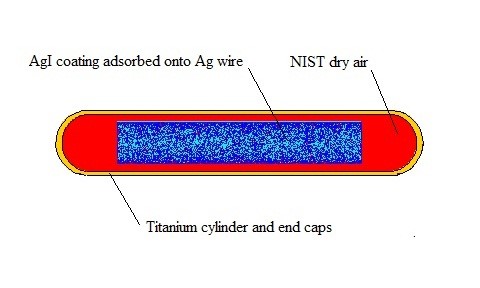

Supplement: Supplementary file 1 — Supplementary Material [file ACM2-16-226-s001.jpg]

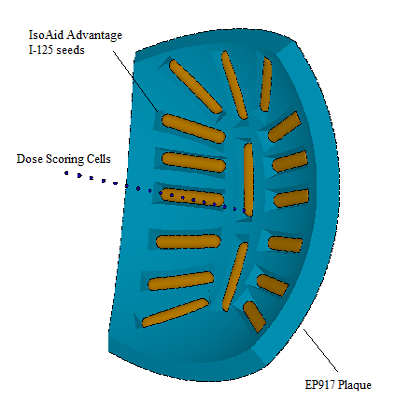

Supplement: Supplementary file 2 — Supplementary Material [file ACM2-16-226-s002.png]
